# An Evaluation of Root Phytochemicals Derived from* Althea officinalis* (Marshmallow) and* Astragalus membranaceus* as Potential Natural Components of UV Protecting Dermatological Formulations

**DOI:** 10.1155/2016/7053897

**Published:** 2016-02-03

**Authors:** Alison Curnow, Sara J. Owen

**Affiliations:** Clinical Photobiology, European Centre for Environment and Human Health, University of Exeter Medical School, Knowledge Spa, Royal Cornwall Hospital, Truro, Cornwall TR1 3HD, UK

## Abstract

As lifetime exposure to ultraviolet (UV) radiation has risen, the deleterious effects have also become more apparent. Numerous sunscreen and skincare products have therefore been developed to help reduce the occurrence of sunburn, photoageing, and skin carcinogenesis. This has stimulated research into identifying new natural sources of effective skin protecting compounds. Alkaline single-cell gel electrophoresis (comet assay) was employed to assess aqueous extracts derived from soil or hydroponically glasshouse-grown roots of* Althea officinalis* (Marshmallow) and* Astragalus membranaceus*, compared with commercial, field-grown roots. Hydroponically grown root extracts from both plant species were found to significantly reduce UVA-induced DNA damage in cultured human lung and skin fibroblasts, although initial* Astragalus* experimentation detected some genotoxic effects, indicating that* Althea* root extracts may be better suited as potential constituents of dermatological formulations. Glasshouse-grown soil and hydroponic* Althea* root extracts afforded lung fibroblasts with statistically significant protection against UVA irradiation for a greater period of time than the commercial field-grown roots. No significant reduction in DNA damage was observed when total ultraviolet irradiation (including UVB) was employed (data not shown), indicating that the extracted phytochemicals predominantly protected against indirect UVA-induced oxidative stress.* Althea* phytochemical root extracts may therefore be useful components in dermatological formulations.

## 1. Introduction

Skin cancer incidence is known to have increased significantly in the last 20 years, despite nonmelanoma skin cancer (NMSC) being greatly under reported [1–4]. As lifetime exposure to ultraviolet radiation (UV) has risen, the health effects have also become more apparent particularly within older populations (60 years of age plus) [5]. More than 70% of all skin cancer cases presenting in this age group are NMSC, which are primarily thought to be caused by excess UV exposure accumulated over time [6]. Although NMSC is rarely fatal, its morbidity is significant and treatment often places a significant burden on healthcare provision. Exposure to sunlight is not entirely avoidable or indeed desirable however, as it is also necessary for essential biological functions to occur (i.e., vitamin D metabolism) [7].

UV radiation consists of three wavelength ranges UVA (315–400 nm), UVB (280–315 nm), and UVC (<280 nm). Of these, mainly UVA and UVB are of physiological importance as UVC is absorbed by oxygen and ozone in Earth's atmosphere [8, 9]. Acute effects of overexposure of the skin to UV manifest as erythema (sunburn), whereas chronic effects can develop into skin cancer or lead to premature photoageing [10]. The involvement of UV as the major causal factor in the aetiology of skin cancer is very persuasive and has arisen from extensive animal studies and the effect of solar radiation on genetic mutation [6, 11]. UVB radiation has sufficient energy to directly damage DNA by inducing base modifications such as cyclobutane pyrimidine dimers (CPDs), 6-4 photoproducts (6-4PPs), and thymidine glycols [11–16]. CPDs are generally the more abundant lesion type leading to cytotoxicity, with 6-4PPs being less represented but potentially more mutagenic. Lower energy UVA can penetrate deeper into the skin than UVB and causes indirect DNA damage through the activation of reactive oxygen species (ROS). These genotoxic reactions induce single strand breaks (SSBs) in DNA, DNA-protein cross-linking, or oxidisation of bases [17]. There is also an increasing evidence from both animal and human* in vitro* studies that UVA irradiation has a more significant role in skin carcinogenesis than previously thought [18–24]. Historically, UVA-induced carcinogenesis has been attributed to oxidative DNA base modification such as 7,8-dihydro-8-oxoguanine (8-OHG) [25, 26]. More recent studies have indicated that, along with 8-OHG, pyrimidine dimers are a major contributor in UVA mutagenesis particularly CPDs at cytosine-dipyrimidine sites [27–29]. It is speculated that a weak activation of p53 following UVA exposure may be more mutagenic than UVB exposure as there is increased chance of cell survival with nonrepaired DNA damage, potentially leading to the induction of skin carcinogenesis [24]. This is particularly significant when many modern tanning devices employ the UVA spectrum rather than the UVB [30] and sunscreens predominantly provide protection against the latter with less protection against the DNA damage induced by UVA irradiation being incorporated [31].

The cells of the body, including the skin, have very effective defence mechanisms in place however to protect UV-absorbing nucleic acids and proteins, in particular cellular DNA, from damage [17]. The availability and abundance of these mechanisms (be they physically absorbing or reflecting UV irradiation, scavenging free radicals, or repairing cellular damage) are essential to minimize the potential mutagenic and carcinogenic effects of UVA and/or UVB within the cellular environment [32]. It is impossible however for these defence systems to completely inhibit UV-induced damage and the resulting impact can lead to cell death, senescence, or carcinogenesis [33]. Therefore, there has been a significant effort in recent years to stem the rising incidence of UV-related skin cancer through education programmes [34]. The development of sunscreen products and skincare formulations containing UV protection factors for a range of skin types has also become a prominent feature in the cosmetic industry [31]. Such products are marketed heavily on their ability to prevent sunburn while still allowing the skin to tan, permitting the length of sun exposure time to be increased whilst also suggesting a reduction in the likelihood of developing skin cancer and photoageing. Traditionally, sunscreens were designed to prevent sunburn (UVB-induced erythema), the sun protection factor (SPF) indicating the number of minimal erythema doses (MED) an individual can tolerate before developing erythema. To date, there is no validated measure regarding the protection proffered by sunscreens to indirect skin damage caused by UVA although several methods have been proposed [35–39].

Increasing public demand for dermatological products containing components derived from nature has also increased the desire to identify novel naturally occurring UV protecting compounds that can be employed in such formulations [21, 33, 40–44]. Phytochemicals have been used in herbal medicine and traditional remedies for many years and can have beneficial or detrimental effects depending on their use [44].* Aloe vera* and vitamin E are two of the most prominent naturally derived plant chemicals employed in skin care products. In addition to negating the effects of erythema and inflammation in the skin, phytochemicals may also provide important antioxidant and UV-absorbing properties, which could reduce or prevent the UV-induced DNA damage [45] that may potentially initiate skin carcinogenesis.

An initial general review of the literature indicated that* Althea officinalis* (AL; Marshmallow) and* Astragalus membranaceus* (AS; also known as Huang Qi in traditional Chinese medicine) are just two of the many plant species that have been used in alternative medical therapies for many years (e.g., Chinese medicine) to treat a variety of ailments including digestive disorders, compromised immunity, colds, wound healing, and inflammation. Unlike in other plants, however, in both these particular species the roots are of particular interest as these are thought to contain skin protecting polysaccharides and/or UV-absorbing compounds which may have potential in protecting against solar-induced skin damage. Root extracts of each of these species (AL and AS) have therefore been investigated here to see if they could protect the DNA of cultured human cells from the DNA damage known to be induced by UV irradiation. Additionally, as there is currently a move away from using organic solvents due to their potential toxicity and/or environmental concerns relating to their disposal, the root extracts investigated were prepared by aqueous extraction using supercritical water to increase the hydrophobicity above that of water at normal atmospheric pressure. The alkaline comet assay (single-cell gel electrophoresis) as described by Singh et al. [46] can assess DNA damage in the form of single strand breaks, double strand breaks, and alkali labile sites and so has been employed to assess the effect of these plant-derived compounds (phytochemicals) on protecting the integrity of cellular DNA* in vitro*.

## 2. Materials and Methods

### 2.1. Phytochemical Sample Preparation

In order to be used in a commercial capacity, a pure and consistent source of the phytochemicals was essential. The extracted root material supplied for this study was therefore produced by cultivating each plant species,* Althea officinalis* (AL; Marshmallow) and* Astragalus membranaceus* (AS) separately using carefully maintained hydroponic or soil growing conditions in a glasshouse (by ADAS UK Ltd., UK) as follows.

Seeds of AL and AS were utilised from a single stock supplied by Horizon Herbs LLC (Williams, OR 97544, USA). Plants were raised initially in rock wool plugs and later transplanted to the hydroponic or soil based system. Hydroponic production employed a nutrient film technique (NFT), and plants were placed in net pots at densities of 40 plants/m^2^ for AL and 80 plants/m^2^ for AS. A standard nutrient regime with 124 mg NO_3_ L^−1^ was used for both species, and electrical conductivity and pH were monitored and controlled automatically. Soil-grown plants were grown in peat based compost in growbags in the glasshouse alongside the NFT units, such that both treatments experienced the same light and temperature regimes. Plant density in the growbags mirrored that in the adjacent hydroponic channels. Roots were cut off when the NFT channels became full and the plants were then allowed to re-grow. The soil-grown plants were harvested once at the end of the season, as roots could not be harvested continuously as in the NFT system. Soil-grown roots were washed in a commercial carrot washer to remove compost prior to drying.

The materials grown in the glasshouse were compared to samples of field-grown roots sourced from commercial suppliers [AL; G. Baldwin and Co. (Walworth Rd, London, UK) and/or Base Formula Co. (North Street, Melton Mowbray, UK); AS; G. Baldwin and Co].

The root crop subsequently harvested was dried gently at 40°C in a forced air flow oven until reaching constant temperature before processing using a novel extraction method using superheated water (University of Leeds/Critical Processes Ltd., UK) to optimize the production and extraction of UV protecting molecules and polysaccharides [47]. Ten and a half grams of root material was extracted at 150°C for 120 minutes, collecting 240 mL in 3 continuous 80 mL portions. These samples were labelled extracts 1, 2, and 3, respectively. This was done to see if different phytochemical components were eluted at different time points from the extraction process and if these different fractions possessed different biological activity on subsequent analysis. An equal portion of each of these three timed extract samples was then reserved to form three “whole” extract samples (labelled “W1,” “W2,” and “W3,” resp.) which contained a representation of all the phytochemicals extracted from the plant root over each subsequent period of elution. Further equal portions of extracts 1, 2, and 3 were then subjected to ultrafiltration using disposable in-line membrane filters designed for the use in a laboratory centrifuge. Membranes were chosen with a cut-off of 30 kDa to separate polysaccharides from low molecular weight species. This produced six more samples for analysis, a retentate (labelled “R”) and filtrate (labelled “F”) of each of these three timed extracts.

These processes produced consistent, pure samples of the roots of each plant species ready for testing (or use in a sunscreen product). All samples for comet assay analysis were supplied in phosphate buffered saline (PBS) at various concentrations (mg/mL) and were confirmed to be sterile solutions via microbiological analysis.

### 2.2. Human Fibroblast Cell Culture and Phytochemical Exposure

Initial experiments were conducted using human fetal lung fibroblasts (MRC-5) (ECACC Number 84108101, UK) derived from the normal lung tissue of a 14-week-old male. Human skin fibroblasts (84BR) (ECACC No 90011805, UK) derived from a biopsy of a radiosensitive female were used for subsequent experimentation. Fibroblasts were cultured at 37°C with 5% CO_2_ in Eagles modified medium (EMEM) supplemented with 10 or 15% fetal calf serum (FCS), respectively, 200 mM L-glutamine and 2% penicillin/streptomycin solution (1000 iu penicillin and 1 g streptomycin). All reagents were supplied by Sigma (UK) unless otherwise stated. Monolayers of cells were grown aseptically in 25 cm^2^ vented tissue culture flasks until they were 70% confluent and were then washed twice with PBS to remove the spent medium. Fresh medium was added (10 mL) and supplemented with extract (which had been passed through a 0.22 *μ*m filter to maintain sterility) at a dilution factor of 1 : 100 (as determined from an initial dose escalation experiment conducted over the concentration range of 1 : 10 to 1 : 1000 with MRC-5 lung fibroblasts; data not shown) and incubated for one hour before harvesting (the time selected from previous studies [48]). Cells were detached from the bottom of the culture flasks using 0.25% trypsin/EDTA and centrifuged for 3 minutes at 1500 rpm before suspension in PBS. Cell viability was assessed using trypan blue dye exclusion (>95%) and suspensions diluted to provide 600,000 cells/mL for comet assay analysis.

### 2.3. Alkaline Single-Cell Gel Electrophoresis (Comet Assay)

Alkaline single-cell gel electrophoresis (comet assay), described by Singh et al. [46], can assess DNA damage in the form of single strand breaks, double strand breaks, and alkali labile sites. To assess the efficacy of phytochemicals in the extracts of plant root material, the comet assay was used to determine the level of DNA damage induced by a controlled light insult in the cells incubated in the presence or absence of the test substances. All samples were tested in quadruplicate (60 comets scored per area, 240 comets per sample). The alkaline comet assay was carried out as described in detail by Morley et al. [48]. Briefly, 50 *μ*L of cell suspension was mixed with 500 *μ*L premolten (43°C) 0.5% low melting point agarose (LMP; LMAgarose, AMS, Trevigen Inc., USA). Aliquots (75 *μ*L) of this cell/LMAgarose mixture were then transferred to each of the two circular sample areas of CometSlide*™* glass microscope slides (AMS, Trevigen Inc., USA). Slides were left to set at 4°C for 15 minutes before irradiation. Following irradiation (described below), slides were immediately immersed in lysis solution (AMS, Trevigen Inc., USA) to prevent cellular repair and kept at 4°C for 1 hour. The DNA was allowed to unwind in an alkaline solution (pH > 13) (200 mM EDTA, NaOH) for 1 hour at room temperature before carrying out electrophoresis at (20 V, 275 mA) for 24 minutes. Following electrophoresis, the slides were rinsed with ethanol and then left to dry at room temperature before analysis. The DNA was stained using ethidium bromide (10 *μ*g/mL) and DNA migration (% tail DNA) analysed using a fluorescence microscope connected to specialist image analysis software (comet assay II, Perceptive Instruments, UK).

### 2.4. Light Source and Irradiation

Irradiation was administered using a 200 W xenon-mercury UV light source (Lightningcure L5, Hamamatsu Photonics Ltd., UK) with a four-furcated liquid light guide directed towards test areas on four separate comet slides simultaneously. Test areas were exposed to uniform, stable light intensity within the same wavelength range as that of terrestrial solar radiation, with (UVA + visible irradiation) or without (UVB + UVA + visible irradiation) the presence of a 320 nm cut-on filter (CG-WG-320; Elliot Scientific Ltd., UK) to remove wavelengths below 320 nm. UVA + visible light exposure was carried out for a minimum of 12 minutes, UVA + UVB + visible light exposure for 60 seconds (data not shown). These irradiation levels were determined as those required to reliably initiate sufficient DNA damage (circa 50%) in this test system and observe any significant changes (positive or negative) attributable to the presence of phytochemicals in the extracts being tested.

The potential effects of the presence of antioxidants on cellular DNA in this test system were initially assessed using N-acetylcysteine (NAC; Sigma, UK), a low molecular weight antioxidant linked to free radical scavenging and singlet oxygen quenching as a positive test control substance (data not shown).

### 2.5. Statistical Analysis

Statistical analysis was conducted on median values utilising the nonparametric Mann-Whitney *U* test. Box-whisker plots were produced using SigmaPlot 11.0 to indicate the median (solid line), ±25% of the data (box), and 10% to 90% spread of the data (whisker).

## 3. Results and Discussion

### 3.1. Effects of Hydroponically Grown AL and AS Root Extracts on Human Fibroblasts ± UV Irradiation

**Figure 1:**
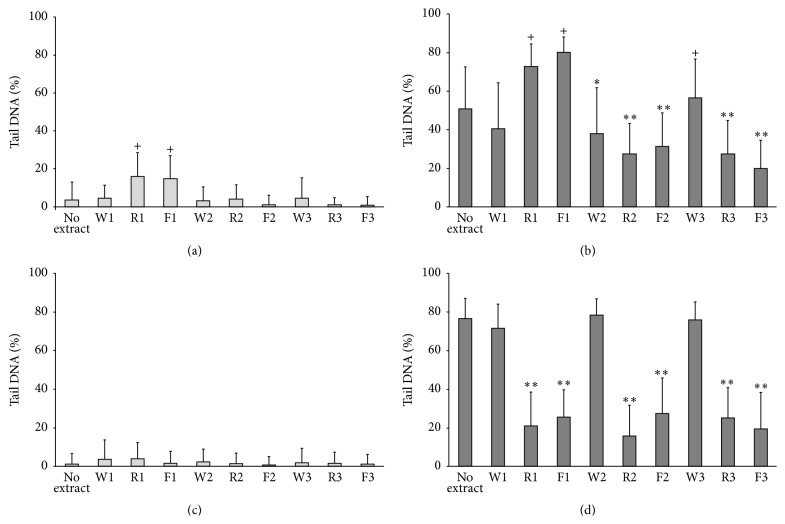
Median percentage DNA damage in the tail of comets derived from cultured human lung fibroblasts exposed to (a)* Astragalus* extracts without irradiation, (b)* Astragalus* extracts with 16 minutes of filtered xenon-mercury irradiation (UVA + visible light), (c)* Althea* extracts without irradiation, and (d)* Althea* extracts with 16 minutes of filtered xenon-mercury irradiation (UVA + visible light). W = whole extract, R = retentate, and F = filtrate collected from elution periods 1, 2, and 3. Bars indicate the 75 percentile of the data set. + indicates a statistically significant increase in damage (*p* < 0.001) when compared with the corresponding control group without extract exposure. *∗* and *∗∗* indicate a statistically significant decrease in damage (*p* < 0.05 and *p* < 0.001, resp.) when compared with the no extract control group.

MRC-5 cells (human lung fibroblasts) were incubated for one hour with or without timed extracts from AL or AS and then they are exposed to either 16 minutes of filtered xenon-mercury irradiation (UVA + visible light) or no irradiation (dark control) (Figure 1). No increase in DNA damage was observed in nonirradiated cells exposed to any of the AL extracts (Figure 1(c)). Only nonirradiated cells exposed to AS-derived extracts R1 and F1 (Figure 1(a)) showed increase in DNA damage (*p* < 0.001). These extracts were also found to increase levels of DNA damage on exposure to UVA irradiation (*p* < 0.001) (Figure 1(b)). These results suggest that there was a genotoxic effect produced in the cells, by the phytochemical compounds contained within the AS extract, prior to irradiation commencing.

With irradiation (Figure 1(b)), AS whole extract W2 (*p* < 0.05) and ultrafiltrated extracts R2, F2, R3, and F3 (*p* < 0.001) significantly reduced UVA-induced DNA damage. The change in DNA damage in cells following UVA irradiation incubated with AS whole extract W1 was not found to be of statistical significance and extract W3 was found to statistically increase the level of DNA damage observed (*p* < 0.001). With AL phytochemical exposure followed by irradiation (Figure 1(d)), all ultrafiltrated extracts (R and F) from each sample collection period considered reduced the UVA-induced DNA damage normally induced by the light insult (*p* < 0.001). So, although it would appear that extracts from the AS species could potentially be effective against UVA exposure, their capacity to induce UVA damage cannot be ignored, particularly if considering its use in emollients in future human studies. Future studies therefore concentrated on* Althea*, whose retained and filtered extracts all significantly reduced the UVA-induced genotoxicity produced in this cell type (Figure 1(d)).

**Figure 2:**
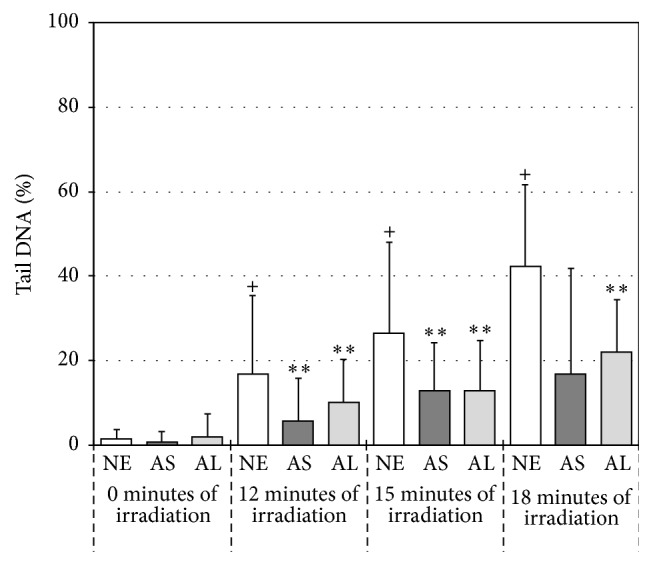
Median percentage DNA damage in the tail of comets derived from cultured human skin fibroblasts incubated with no extract (NE),* Astragalus* extract W2 (AS), or* Althea* extract W2 (AL) for one hour followed by 0, 12, 15, or 18 minutes of filtered xenon-mercury irradiation (UVA + visible light). Bars indicate the 75 percentile of the data set. + indicates a statistically significant increase in damage (*p* < 0.001) when compared with the NE dark control. *∗∗* indicates a statistically significant decrease in damage (*p* < 0.001) when compared with the NE control at the same irradiation period.

Clear and significant reductions in UVA-induced DNA damage were also apparent in the human skin fibroblasts (84BR) when using extracts W2 from both plant species (Figure 2). This was encouraging as these dermatological cells were (as anticipated) less sensitive to the effects of UVA than the lung-derived MRC-5 cells and a more relevant cell type when investigating potential sunscreen/skincare constituents.

Figure 2 demonstrates that with the increasing periods of filtered xenon-mercury irradiation (UVA + visible light) without the presence of any extracts, the % tail DNA damage observed increased (*p* < 0.001) when compared to the dark control. This genotoxic damage was reduced in the presence of whole extract W2 from either AS or AL (*p* < 0.001), although the responses observed with each species at 15 and 18 minutes of exposure were not significantly different from one another (*p* > 0.05). Although these results cannot be directly extrapolated to those of an* in vivo* skin system which has vasculature and immunological factors to consider, there does appear to be target compound or compounds present in the extracts, which is combating the oxidative stress-induced genotoxic damage being produced by UVA radiation. Furthermore, as these positive results were obtained using “whole” root extracts, it appears that the extra processing step of ultrafiltration was not essential for efficacy.

Experimentation was also conducted to consider the effect of UVB on the more robust and UV-sensitive human lung fibroblasts (MRC-5). None of the whole or ultrafiltrated extracts of AL or AS were found to reduce the levels of DNA damage observed using UVB + UVA + visible light with the experimental conditions employed (60 seconds of unfiltered xenon-mercury irradiation; data not shown). This was not unexpected as UVB is able to damage DNA directly and would suggest that either the components of the extracts were not able to absorb the UVB or there was insufficient amount of effective material present. This also indicated that the protection observed against UVA + visible irradiation in Figures 1 and 2 was most likely derived through the prevention of indirectly-induced light-mediated genotoxic damage. In addition, the system was validated prior to investigation of AL and AS extracts using N-acetylcysteine (NAC) (data not shown). So, experimental conditions capable of detecting protection by a known antioxidant compound were employed throughout.

Due to the novel extraction system used, the exact constituents of the test extracts employed and their concentration were unknown. Chemical analysis (conducted by Royal Botanic Gardens, Kew, UK) established that AS extracts contained simple phenolics (caffeic, p-coumaric acids), various flavonoids, isoflavones, and saponins (astragalosides). AL extracts were found to predominantly contain carbohydrates and simple phenolics as well as 8-hydroxyflavones including luteolin (the latter found more so in extract W2). Several unidentified flavonoid-like compounds were also detected.

### 3.2. Effects of Hydroponic, Commercial, or Soil Derived AL Root Extracts on Lung Fibroblasts ± UVA Irradiation

**Figure 3:**
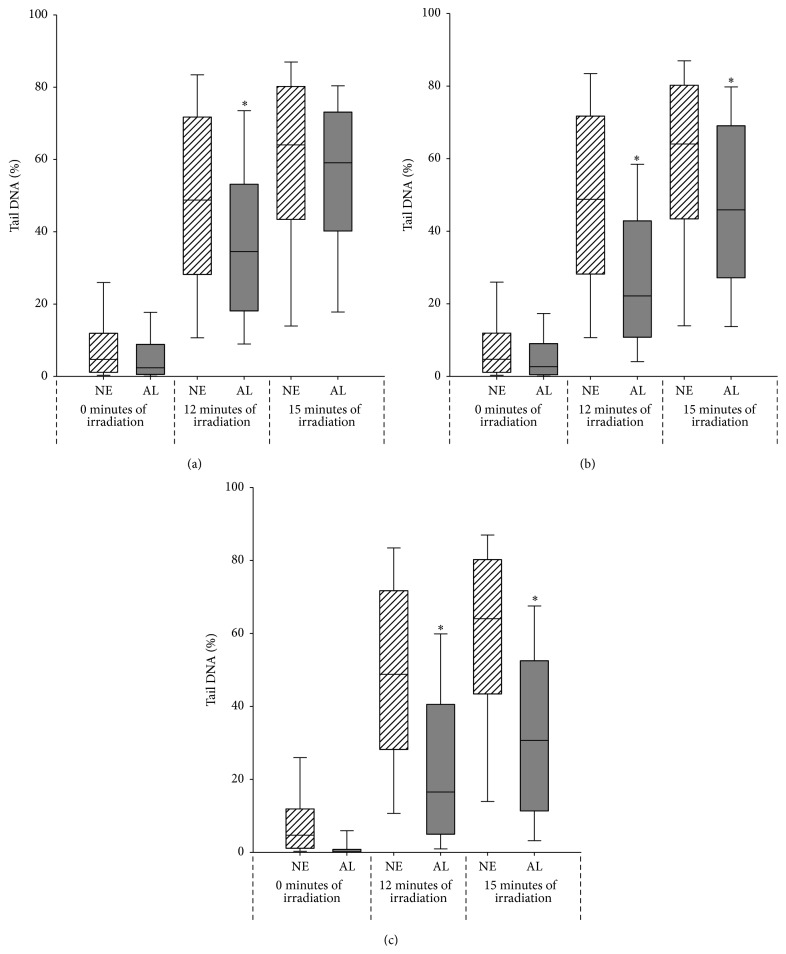
Median percentage DNA damage in the tail of comets derived from MRC-5 cells incubated with no extract (NE) or whole extracts of (a) commercially sourced field-grown, (b) hydroponically glasshouse-grown, or (c) glasshouse soil-grown* Althea* (AL) roots for one hour followed by 0, 12, or 15 minutes of filtered xenon-mercury irradiation (UVA + visible light). Solid bar indicates median, box indicates ± 25% of the data, and the whisker indicates the 10–90% spread of the data. *∗* indicates a statistically significant decrease in damage (*p* < 0.001) when compared with the control group irradiated for the same time period without extract exposure.

Investigation of the efficacy of different AL preparations was carried out to determine if there were differences in the level of UVA protection afforded when the roots were obtained from* Althea* plants that had experienced different growing conditions. Due to the potential genotoxicity of extracts from AS (Figures 1(a) and 1(b)), only AL was deemed suitable for further analysis as a potential candidate for use in a topical dermatological product.  Figure 3 indicates how similar the preparations of commercially sourced field-grown (Figure 3(a)), hydroponically glasshouse-grown (Figure 3(b)), or glasshouse soil-grown (Figure 3(c)) derived AL extracts were in their effect on the levels of UVA-induced DNA damage in human lung fibroblasts. Cells were exposed to 0 (dark control), 12 or 15 minutes of filtered xenon-mercury irradiation (UVA + visible light).

Increasing periods of irradiation without extract incubation resulted in increased DNA damage (*p* < 0.001). All AL extracts (independent of source) significantly reduced the effect of UVA-induced DNA damage with 12 minutes of UVA + visible light (*p* < 0.001) (Figure 3). Hydroponically derived extract (Figure 3(b)) continued to significantly reduce DNA damage up to 15 minutes (*p* < 0.001) as did the glasshouse soil-grown root extract (Figure 3(c)) (*p* < 0.001), although in each case the protection afforded diminished with the continued light exposure. The extract from the commercially derived* Althea* roots offered the least period of protection (Figure 3(a)). These results appear to indicate the presence of similar components in each of the extracts with the most potent being in that of the glasshouse-grown, soil derived AL sample. This is quite possible as different growing conditions may affect the levels of particular phytochemicals and thus the potency of the extracts. The lower activity of the commercially derived field-grown AL extract could also be due to the effects of processing during manufacture, reducing the potency or concentration of the effective compound. Additionally, the glasshouse-grown materials were cultivated from seeds of a particular genetic stock, whereas this was an unknown quantity with the commercially sourced material.

## 4. Conclusions

Hydroponically grown root extracts from both plant species investigated were found to significantly reduce UVA-induced DNA damage in cultured human lung and skin fibroblasts, although initial AS experimentation detected some genotoxic effects, indicating that AL root extracts may be better suited as potential constituents of dermatological formulations. Glasshouse-grown soil and hydroponic AL root extracts also afforded cultured human cells with statistically significant protection against UVA irradiation for a greater period of time than the commercial field-grown roots, indicating that these systems of cultivation may convey beneficial effects (for instance in terms of antioxidant content) over and above that achieved via more traditional growing methods. No significant reduction in DNA damage was observed when total ultraviolet irradiation (including UVB) was employed, indicating that it is most likely that the extracted phytochemicals predominantly protected against indirectly produced UVA-induced oxidative stress. This factor could be considered in more detail within future experimentation employing the enzyme formamidopyrimidine DNA glycosylase (FPG) to modify the comet assay protocol to reveal oxidised bases.

From the point of view of preventing photoageing and/or potential skin carcinogenesis, the inclusion of such compounds in formulations designed to protect the skin may with further investigation prove to be beneficial. The conclusions that can be drawn from the data presented here in this particular respect, however, are somewhat limited. This is because the alkaline comet assay is only detecting genotoxic DNA damage and does not indicate where in the genome the damage is occurring or whether this damage may potentially be mutagenic or carcinogenic. It cannot therefore be concluded definitively that by preventing this genotoxic damage with phytochemical containing root extracts this would prevent or reduce cancer development, although this may be feasible. Additionally the cells were lysed immediately following light irradiation and so they were given no opportunity to repair the light-induced damage sustained or alternatively to trigger apoptotic cell death, and future investigations should consider these aspects.

So, in conclusion, this investigation has demonstrated that phytochemical containing root extracts do have the potential to be useful natural components in dermatological formulations where a reduction in oxidative stress-induced damage is desired, with the glasshouse-grown soil derived AL roots producing the greatest level of protection against UVA-induced DNA damage observed. Additionally, more extensive chemical analysis of the extracts may be able to identify the individual phytochemical effector(s) involved in the protection afforded by these plants and further research may indicate whether these compounds do indeed have the potential to prevent some of the carcinogenetic processes known to be induced by sunlight.
